# Notes and News

**Published:** 1886-12-25

**Authors:** 


### Notes and News.

Collected and Communicated.

Between £1,300 and £1,400 were obtained from the bazaar held recently in Kensington, and the supplementary sale in Canonbury, for the funds of the Great Northern Central Hospital.

At a special meeting of the Committee of the Leeds Public Dispensary, a resolution was unanimously passed cordially thanking the Committee, Treasurer, and Secretary of the Leeds Musical Festival for the sum of £390, which had been allotted to the Dispensary.

Mr. John North has presented the Leeds Infirmary and Cookridge Convalescent Hospital with £4,000, for the purpose of erecting a building on the Cookridge grounds to accommodate the semi-convalescent patients of the Infirmary.

A somewhat novel request has been made by the authorities of the Royal South Hants Infirmary, who state that medicine-bottles are much needed, and that any person having a number of no further use to himself will confer a benefit on the institution by sending them there.

Mr. T. M. Hall has been selected, out of ten candidates for the appointment, as secretary to the Ingham Infirmary, South Shields. The salary is £30 per annum, in addition to five per cent. on all subscriptions collected, with the exception of sums contributed by the working-classes of the district.

In alluding lately to the opening of the Workington Infirmary by Mrs. Wilson, we omitted to mention that that lady's husband, Mr. Alexander Wilson, had kindly sent a cheque for £380, to enable the committee to open the institution free of debt. We have pleasure in recording this beneficent act.

The *Scottish News* of the 4th inst. publishes a letter from a correspondent, suggesting that dictionaries in various languages should be provided for the use of the inmates of the Edinburgh Royal Infirmary, many of whom are foreigners.

At the twenty-fourth annual meeting of the Surgical Aid Society, held at the Cannon Street Hotel on Dec. 6th, it was announced that the Earl of Aberdeen and the Lord Mayor (Sir Reginald Hanson) have accepted the posts of President and Vice-President of the institution respectively. During the past year the Society has relieved 6,412 persons, or an average of 123 per week.

The name of Mr. John Fleming has been added to the list of trustees of the proposed Fleming Memorial Hospital for Sick Children at Newcastle-upon-Tyne; the plans of which have now been prepared by Messrs. Quilter and Wheelhouse, of 10, Brunswick Square, W.C. The building will be of red brick with stone facings, and will be provided with over sixty beds; the cost, irrespective of the site, is estimated at £15,000.

The last collection of the present year at the Burnley mills and workshops, in aid of the maintenance fund of the Victoria Hospital, has realised nearly £200. The hospital was opened for the reception of in-patients on the 6th inst. We are glad to learn that the institution is not only free from debt, but that there is a large sum in hand towards next year's expenses.

The new hospital in Jervis Street, Dublin, is to be opened on New Year's Day, and a ball, in aid of the maintenance fund, will be given on the 19th January. We understand that the delay in opening the institution has been due to want of funds to pay the contractor, but this difficulty is now happily settled.

The four-days' bazaar lately held in Bradford, in aid of the extension fund of the Hospital for Sick Children, has been a magnificent success, the total receipts amounting to no less than £5,070. The inhabitants of Bradford have indeed set a splendid example of generous philanthropy to their fellow-subjects throughout the country.

Mr. John Henry Lloyd, Hon. Sec. of the Birmingham Children's Hospital, appeals for gifts of toys, fruit, warm clothing, etc., for the benefit of the little inmates of that establishment. Coming as it does at a time of year which the children of all classes look forward to as one of especial happiness, we trust this appeal will meet with a warm response.

A "Hospital Medical Officer," writing to the *Sheffield Independent* of the 11th Dec., suggests that the growing practice among well-to-do people, of obtaining treatment at charitable institutions, should be stopped by the prosecution of the would-be patients, whom he stigmatises as swindlers. "In one case, that I discovered," he says, "I sent in a bill for the attendance, as though the patient had come to me as a private one, and the amount was sent to me at once, without a word of comment. Probably the lady was too ashamed of herself to attempt excuse." This plan seems to us worth following in cases where the imposture can be found out.

Mr. Edward Rhys Wingfield, of Barrington Park, has sent a cheque for 200 guineas to the executive committee of the proposed General Hospital at Merthyr Tydfil, and has also intimated his intention of becoming an annual subscriber to the maintenance of the proposed institution, in due course.

The story which our Special Commissioner told recently of a London Hospital out-patient whom he named the "Artist's Model," has elicited a warm-hearted response from a gentleman who has volunteered to help the family out of their present difficulties. Unfortunately, our Commissioner did not at the time take a note of the girl's address, and the name he cannot recall, the conversation he reported having taken place in August last. If the girl or any friend of hers should see this note, we shall be exceedingly pleased to be the means of putting her into communication with the generous sympathiser who has addressed us.

Various tradesmen having placed several valuable presents at the disposal of the North West London Hospital, the Committee of that establishment have announced their intention of instituting a lottery, with ten-shilling tickets, for these articles. It is difficult to know what other use could be made of such presents, but the example is hardly one to be followed or commended. We are requested to point out that it is not the Great Northern Central Hospital, in the Caledonian Road, as some persons have thought, which is resorting to such unusual methods of raising funds.

The following vacancies are announced this week, the amount of the salary being placed in each case after the name of the institution :—

**Resident Medical Officers.**

Hospital for Insane, Barnwood, Gloucester. Junior Assistant. Doubly qualified.—£100, with board.
Exeter Asylum. Assistant.—£100, with board.

**Non-Resident Medical Officers.**

National Dental Hospital. House Surgeon.—£50.

**Matrons.**

Caterham Cottage Hospital.—£30, and all found.
Hereford General Infirmary.—£70, with board.

**Trained Nurses.**

Trained Nurses' Home, Truro.—Several.
Hospital for Women, 222, Marylebone Road. One.—All found.
Dorset County Hospital. Night.—£25, with board.
Margate Infirmary. Night.—£20, and uniform.
Bridgewater Infirmary. One medical and surgical.

Mr. Francis O'Farrell, writing from Great Yarmouth to the *Freeman's Journal* about the controversy now raging concerning Mercer's Hospital, Dublin, says, "It" (the hospital) "is always hopelessly in debt, filthy, dirty, and neglected, and deserted by students. When recently in Dublin, a fellow-student and I witnessed the interesting performance of a ward-maid scraping the dirt off the staircase with an iron spoon. About the same time, I heard Mr. O'Grady order a nurse to pull down the blind to shade the light from the eyes of a patient whose thigh he had just amputated, and she replied, 'I can't, sir, for there is no blind to pull down;' but the roller was there." Such a state of things is a crying disgrace, and we wonder the people of Dublin do not peremptorily insist upon better provision being made for the sick poor among their fellow-citizens.

The Misses Eastwood, of Newhouse, Huddersfield, have promised £500 towards the building extension fund of the Huddersfield Infirmary, and further sums of £100 and £55 respectively, have already been received from the executors of the late Mr. Richard Wheatley, and the Committee of the Holiday Charities Cup Competition.

A home for convalescents is about to be established at Sidney House, Trewithen Road, Penzance, by Mr. T. Robins Bolitho. The site is well chosen, and admirably adapted to the purpose referred to, being only about two minutes' walk from the public baths and the promenade.

The Liverpool Northern Hospital has been for some time possessed of a Horse Ambulance Service, a boon of inestimable value to its patients ; and we learn that the Royal Southern Hospital will shortly be presented with a similar gift by the friends of the charity.

"Mony a little maks a muckle," and we are glad to learn that a sum of £5 4*s.* 3*d.* has been collected, chiefly in pence, by the school authorities of the parish of Woodchurch, Kent, in aid of the Canterbury Hospital. The circular issued by Canon Routledge some months ago, suggesting that collections should be undertaken by the schoolmasters and schoolmistresses in the different parishes, has, therefore, borne fruit in Woodchurch at any rate.

During the past year, fifty-seven patients have been admitted to the Willingham Cottage Hospital, of whom forty-three have been discharged cured, and six relieved. Since the building was erected in 1880 in memory of the late Rev. W. Reynard, at the expense of Mrs. Reynard, over 300 cases have received treatment within its walls, and out of that number only four deaths have occurred.

The Executive of the Lincoln County Hospital Saturday Fund recently held a meeting in the Albert Hall, Gainsborough. The Bishop of Lincoln, the Right Reverend Dr. King, was present, and there was a large attendance of the general public. It was stated that the collections had realized the following amounts, cheques for which were then handed over to the treasurer of the Hospital, and duly acknowledged by the Rev. H. W. Hutton on behalf of the Governors :—Gainsborough, £103 9*s.* 1½*d.*; Sleaford, £105 ; Lincoln, £300.

The Hospital Saturday collections in Wolverhampton took place on the 12th inst., and realized a total sum of nearly £1,200. A year ago the amount obtained from this source was £1,161.

A Cottage Hospital in commemoration of Ripon Millenary is to be established in that city. The Governors of the local dispensary have a building at their disposal which they have offered for the purpose, and donations of £200 each have been promised by the Dean of Ripon and Mr. R. Kearsley.

At the annual meeting of subscribers to the Royal Albert Hospital, Devonport, held on the 30th Nov., the report for the past year was read by the Secretary, Mr. C. A. Shapcote, from which it appears that the average daily number of patients under treatment has decreased from 76 in 1884-5, to 71 in 1885-6. The average cost of maintenance of each occupied bed was £47 10*s.* 7*d.* One noteworthy fact mentioned in the report, and about which regret is expressed by the Committee, is the want of interest in, or rather of desire to take advantage of, the pay-wards in the Royal Albert Hospital, only two patients having been treated in these wards throughout the past twelve months.

A bazaar and fancy fair was opened on the 24th ult at the Earlsfort Terrace Rink, Dublin, in aid of the Adelaide Road Children's Hospital, which requires new premises. The committee had in hand for this purpose about £800, but as £700 more was needed, the bazaar was organised, in the hope of obtaining it, under the patronage of the Duke and Duchess of Connaught,

Princess Edward of Saxe Weimar, the Marchioness of Londonderry, and many well-known ladies. A novel feature, and one which attracted much attention, was a "Hospital Stall," which was built in imitation of a Swiss cottage, covered with snow and surrounded by a forest of mimic pine-trees. At night it was lighted inside, and an artificial moon shone down upon it from the roof, producing a singularly pleasing effect. The ladies in charge of this stall wore the dress of nursing sisters of the Society of St. John of Jerusalem, and several little boys, dressed as Swiss peasants, aided them in their labours.

---

A PLEASING incident occurred at the last monthly entertainment given to the in-patients of St. John's Hospital for Diseases of the Skin, held at the branch at Markham Square, Chelsea, when Mrs. Mercier, the wife of Mr. St. Vincent Mercier, the treasurer of the institution, was presented with a most lovely bouquet by the grateful sufferers. The little present came as a perfect surprise to the lady, who was evidently touched at this mark of appreciation of her services in organising these excellent entertainments.

